# *Vibrio vulnificus* MARTX cytotoxin causes inactivation of phagocytosis-related signaling molecules in macrophages

**DOI:** 10.1186/s12929-017-0368-2

**Published:** 2017-08-19

**Authors:** Chun-Liang Chen, Shu-Chun Chien, Tzeng-Horng Leu, Hans I-Chen Harn, Ming-Jer Tang, Lien-I Hor

**Affiliations:** 1 Department of Microbiology and Immunology, College of Medicine, National Cheng Kung University, Tainan, 70101 Taiwan; 2 Institute of Basic Medical Sciences, College of Medicine, National Cheng Kung University, Tainan, 70101 Taiwan; 3 Department of Pharmacology College of Medicine, National Cheng Kung University, Tainan, 70101 Taiwan; 4 Department of Physiology, College of Medicine, National Cheng Kung University, Tainan, 70101 Taiwan

**Keywords:** *Vibrio vulnificus*, MARTX, Antiphagocytosis, Cell rounding, Signaling pathway, Domain-deletion mutants

## Abstract

**Background:**

*Vibrio vulnificus* is a marine bacterial species that causes opportunistic infections manifested by serious skin lesions and fulminant septicemia in humans. We have previously shown that the multifunctional autoprocessing repeats in toxin (MARTX_Vv1_) of a biotype 1 *V. vulnificus* strain promotes survival of this organism in the host by preventing it from engulfment by the phagocytes. The purpose of this study was to further explore how MARTX_Vv1_ inhibits phagocytosis of this microorganism by the macrophage.

**Methods:**

We compared between a wild-type *V. vulnificus* strain and its MARTX_Vv1_-deficient mutant for a variety of phagocytosis-related responses, including morphological change and activation of signaling molecules, they induced in the macrophage. We also characterized a set of MARTX_Vv1_ domain-deletion mutants to define the regions associated with antiphagocytosis activity.

**Results:**

The RAW 264.7 cells and mouse peritoneal exudate macrophages underwent cell rounding accompanied by F-actin disorganization in the presence of MARTX_Vv1_. In addition, phosphorylation of some F-actin rearrangement-associated signaling molecules, including Lyn, Fgr and Hck of the Src family kinases (SFKs), focal adhesion kinase (FAK), proline-rich tyrosine kinase 2 (Pyk2), phosphoinositide 3-kinase (PI3K) and Akt, but not p38, was decreased. By using specific inhibitors, we found that these kinases were all involved in the phagocytosis of MARTX_Vv1_-deficient mutant in an order of SFKs-FAK/Pyk2-PI3K-Akt. Deletion of the effector domains in the central region of MARTX_Vv1_ could lead to reduced cytotoxicity, depending on the region and size of deletion, but did not affect the antiphagocytosis activity and ability to cause rounding of macrophage. Reduced phosphorylation of Akt was closely associated with inhibition of phagocytosis by the wild-type strain and MARTX_Vv1_ domain-deletion mutants, and expression of the constitutively active Akt, myr-Akt, enhanced the engulfment of these strains by macrophage.

**Conclusions:**

MARTX_Vv1_ could inactivate the SFKs-FAK/Pyk2-PI3K-Akt signaling pathway in the macrophages. This might lead to impaired phagocytosis of the *V. vulnificus*-infected macrophage. The majority of the central region of MARTX_Vv1_ is not associated with the antiphagocytosis activity.

**Electronic supplementary material:**

The online version of this article (doi:10.1186/s12929-017-0368-2) contains supplementary material, which is available to authorized users.

## Background

*Vibrio vulnificus* is a gram-negative bacillus distributed worldwide in estuaries. Strains of this species are currently divided into biotypes 1, 2 and 3 based on their biochemical traits and host range [1, 2]. Biotype 1 *V. vulnificus* comprises most of the clinical and environmental isolates, and may cause serious skin lesions and/or fulminant septicemia in humans contracting this organism via wounds or ingestion of contaminated seafood [3]. Most patients have underlying diseases, particularly chronic liver disorders, and the mortality rate may exceed 50% [3]. A number of virulence factors have been identified in *V. vulnificus*, including capsular polysaccharides [4], iron-acquisition ability [5, 6], flagellum [7], type IV pili [8], extracellular insulin-degrading enzyme [9] and an RTX (repeats in toxin) cytotoxin [10–12].

The RTX toxins are produced by a variety of gram-negative bacterial pathogens, and are characterized by large size (over 100 kDa), the glycine and aspartate-rich (GD-rich) nonapeptide repeats and secretion by the type I secretion system [13]. Like that of *V. cholerae*, the RTX of *V. vulnificus* has multiple effector domains and can undergo autoprocessing, and therefore is a member of multifunctional autoprocessing RTX (MARTX) family [14]. MARTXs contain two conserved repeated motifs, the GD-rich repeats at C-terminus and the repeats at N-terminus, as well as the effector domains in the central region. It has been demonstrated recently that the C- and N-termini of the MARTX in either *V. cholerae* or *V. vulnificus* are required for toxin secretion and effector translocation [15, 16]. In addition, deletion of the effector domains of the MARTX in *V. vulnificus*, which causes necrotic death of a variety of eukaryotic cells [10, 12, 17, 18], abolishes the ability to cause rounding, but not lysis, of HeLa cells [16].

The Rho inactivation domain (RID) implicated in causing host cell rounding [19, 20] is present in most of the MARTXs of biotype 1 *V. vulnificus* [21, 22]. Other domains, like the actin-crosslinking domain (ACD), *Pseudomonas aeruginosa* ExoY-like adenylate cyclase (ExoY), cysteine protease domain (CPD), alpha-beta hydrolase (ABH), the Makes caterpillars floppy-like (MCF) and Ras/Rap1-specific endopeptidases (RRSPs) domains are found in the MARTXs of *V. cholerae* or *V. vulnificus* [21, 22]. These effector domains have been demonstrated to exert various effects in the host cells [19, 20, 23–28]. The DUF1 (domain of unknown function 1) domain has also been recently shown to interact with prohibitin 1 in HeLa cells to induce cytotoxicity [29]. Although the MARTXs of biotype 1 (MARTX_Vv1_) and biotype 2 *V. vulnificus* vary in the compositions of effector domains [21], they both promote the survival of this bacterial species in mice during infection by protecting the organism from engulfment by the phagocytes [10, 11]. However, it is not clear how the MARTX of *V. vulnificus* interferes with ingestion of this microorganism by the phagocytes.

Phagocytosis is an actin-dependent process beginning with engagement of the receptors, like the scavenger receptors, complement receptors, immunoglobulins receptors and toll-like receptors (TLRs), on the phagocyte by bacterial surface components. This causes clustering of the receptors to activate various signaling pathways that lead to actin rearrangement for internalizing the bound bacterium [30]. Actin rearrangement in phagocytosis is known to be regulated by a number of kinases, such as Src family kinases (SFKs) [31], focal adhesion kinase (FAK) [32], proline-rich tyrosine kinase 2 (Pyk2) [32], phosphoinositide 3-kinase (PI3K) [33], Akt [34] and p38 mitogen-activated protein (MAP) kinase [35].

To elucidate the molecular mechanism of the MARTX_Vv1_-mediated prevention of bacteria engulfment by the phagocyte, we compared between a wild-type (WT) *V. vulnificus* strain and its MARTX_Vv1_-deficient (MD) mutant for a variety of phagocytosis-related responses they induced in mouse macrophages. We found that in the presence of MARTX_Vv1_, the macrophage rounded up and lost the ability to internalize the bacteria shortly after infection due to F-actin disorganization. We further examined whether this was associated with inactivation of the signaling molecules involved in induction of phagocytosis. We also isolated a variety of MARTX_Vv1_ mutants with deletions in the effector domains to determine the roles of these domains in the MARTX_Vv1_-mediated antiphagocytosis and cytotoxicity. Our data suggest that MARTX_Vv1_ might inhibit phagocytosis by interfering with activation of signaling molecules involved in induction of phagocytosis, a mechanism independent of cell lysis.

## Methods

### Bacterial strains, cells, culture media and reagents

Strain YJ016, a biotype 1 *V. vulnificus* clinical isolate, and its MARTX_Vv1_-deficient (MD) mutant, HL128 [10], were cultured in LB broth. The mouse peritoneal exudate macrophages (mPEM) was isolated from thioglycollate (Merck)-treated peritoneal cavity of 6 to 8 week-old male BALB/c mice (purchased from the Animal Center of National Cheng Kung University) as described [36]. This experiment was conducted in strict accordance with good animal practice defined in “A guidebook for the care and use of laboratory animals” published by the Council of Agriculture, Executive Yuan in Taiwan. RAW 264.7 cells (mouse macrophage cell line; ATCC® TIB71™), HeLa cells (human cervical carcinoma cell line; ATCC^®^ CCL-2™) and mPEM were cultured in high glucose Dulbecco's Modified Eagle's Medium (DMEM; Gibco) supplied with 10% fetal bovine serum (Caisson), 2 mM _L_-glutamine (Gibco) and 1% penicillin/streptomycin (Caisson). In the bacterium-cell coincubation experiments, the cells were cultured in serum-free high glucose DMEM. PP2 was purchased from Calbiochem. PF-431396, LY294002, Akt1/2 kinase inhibitor, cytochalasin D and dimethyl sulfoxide were purchased from Sigma.

### Isolation of the Δ*vvhA* and MARTX_Vv1_ domain-deletion mutants

The Δ*vvhA* mutant as well as the single, double and triple MARTX_Vv1_ domain-deletion mutants, each contains in-frame deletion(s) of almost the entire target effector domain in MARTX_Vv1_, was isolated by in vivo allelic exchange [37]. The upstream and downstream regions flanking *vvhA* and the MARTX_Vv1_ effector domains to be deleted were amplified from *V. vulnificus* YJ016 by PCR with the primer pairs listed in Table 1. The deletions were detected by PCR and confirmed by DNA sequencing.

**Table 1:** List of bacterial strains and primers

Name	Description ^a^	Note ^b^	Reference/Source
Bacterial strain			
YJ016	wild-type strain		[37]
HL128	MARTX_Vv1_-deficient mutant		[10]
CJ189	Δ*u1* mutant		This study
CJ427	Δ*rid* mutant		This study
CJ338	Δ*u2* mutant		This study
CJ340	Δ*cpd* mutant		This study
CJ355	Δ*gd* mutant		This study
CJ433	Δ*u1*Δ*rid* mutant		This study
CJ190	Δ*u1*Δ*u2* mutant		This study
CJ517	Δ*rid*Δ*u2* mutant		This study
CJ429	Δ*u1*Δ*rid*Δ*u2* mutant		This study
KV105	Δ*vvhA* mutant		[56]
KV188	HL128 Δ*vvhA* mutant		[56]
CJ525	CJ433 Δ*vvhA* mutant		This study
CJ527	CJ529 Δ*vvhA* mutant		This study
Primer			
DU1F1	5′-TGTCGACGGGTCACAAAGTC-3’	Δ*u1*	
DU1R1	5′-AGGATCCAGACGCAGTGGTTGGCAC-3’	Δ*u1*	
DU1F2	5′-AGGATCCGCCGATACGCTGGTTGAGTTG-3’	Δ*u1*	
DU1R2	5′-TGAGCTCAGAAAGCCCTGCGAAGATCG-3’	Δ*u1*	
SC01	5′-AGAGCTCCTGGTTGAGTTGGATGTG-3’	Δ*rid*	
SC02	5′-ATCTAGAACTCGGCTTCCAGATGTA-3’	Δ*rid*	
SC03	5′-ATCTAGACACACATGGCGACCTAAG-3’	Δ*rid*	
SC04	5′-AAGCATGCTACCGCTGCTTGCTCTGC-3’	Δ*rid*	
DF1	5′-TTAGTCGACGTGTTTGGACGCCGACAGAG-3’	Δ*u2*	
DR1	5′-AGGATCCTTCTACCGCTGCTTGCTCTGC-3’	Δ*u2*	
DF2f4	5′-AGGATCCGTTGTTGTGACTCCGACAGC-3’	Δ*u2*	
DR2	5′-TGAGCTCCTGCTGCTCTGATCCAAACC-3’	Δ*u2*	
SC07	5′-AGAGCTCGGGTTGTTGAGTAAAGCG-3’	Δ*cpd*	
SC08	5′-ATCTAGAGCCACTCAAACTGTCCTT-3’	Δ*cpd*	
SC09	5′-ATCTAGAAACGGTATTGCGGAAGGC-3’	Δ*cpd*	
SC10	5′-AAGCATGCACCCGCTAACTGCCCAAG-3’	Δ*cpd*	
JL173	5′-TATGAGCTCCAACGCACCCTTCGGTTG-3’	Δ*gd*	
JL174	5′-GCGTCTAGATAATATTCACCTTCCATT-3’	Δ*gd*	
JL175	5′-GCGTCTAGACGGCAGGTTAAGCGAGTT-3’	Δ*gd*	
JL176	5′-TATGAGCTCTTGGTTGAACTGGACTCG-3’	Δ*gd*	
UVVA0965F	5′-AAATGTCGACACCCACATTAA-3’	Δ*vvhA*	
UVVA0965R	5′-AGAGAAAGCTTAAACAGAGTCAT-3’	Δ*vvhA*	
DVVA0965F	5′-TCCCAAGCTTCCCACATTACAAC-3’	Δ*vvhA*	
DVVA0965R	5′-GGAAGAGCTCACCAAACCCG-3’	Δ*vvhA*	
ERM-1 new	5′-AACATATGGATCAAACTCAAGCCCCG-3’	ERM	
ERM-2 new	5′-AACTCGAGGGTCCCTTTGGCATCATT-3’	ERM	

^a^ *u1, rid, u2, cpd* and *gd* correspond to the U1 region, RID domain, U2 region, CPD domain and GD-rich domain, respectively. The underlined sequences in the primers are the restriction sites introduced

^b^The MARTXVv1 domain-deletion mutant that the primer was used to isolate or the recombinant peptide that the primer was used to clone

### Phagocytosis assay

RAW 264.7 cells were coincubated with the bacteria at a multiplicity of infection (MOI) of 10 for 90 min. The internalized bacteria were then envisualized by acridine orange-crystal violet stain as described [38], and observed under an inverted fluorescence microscope (Olympus IX70). Gentamicin protection assay was used to count the internalized bacterial number. Briefly, the extracellular *V. vulnificus* was killed by gentamicin (100 μg/ml, Gibco) for 30 min, and then the intracellular bacteria released from the cells lysed by 0.5% Triton X-100 were enumerated by plate counts.

### Cytotoxicity assay

The cells and bacteria were coincubated at MOI 10, and the cytotoxicity was then estimated by measuring the amount of lactate dehydrogenase (LDH) released from the lysed cells with the CytoTox96^®^ Non-Radioactive Cytotoxicity Assay kit (Promega).

### Cell rigidity analysis and immunofluorescence microscopy of F-actin

RAW 264.7 cells and bacteria were coincubated on a 12 mm cover glass at MOI 10 for 90 min. The rigidity of each of 20–30 selected cells was then examined by atomic force microscopy (AFM; JPK NanoWizard^®^II). Force-distance curves were plotted to quantify the force needed to indent the membrane for a given distance between the AFM tip and cell surface. The elastic (Young’s) modulus was calculated from the force-distance curves by a modified Hertz model (JPK Instruments) built into Elasticity Fit processing software [39]. To examine F-actin, the cells were washed after coincubation with the bacteria, fixed by 1% formaldehyde, permeablized by 0.5% triton X-100, stained by Alexa Fluor^®^ 488 conjugated phalloidin (Invitrogen), and then observed under a fluorescence microscope (Olympus DP 72).

### Anti-MARTX_Vv1_ antiserum preparation

The primer pair used to amplify the ERM domain of MARTX_Vv1_ (indicated in Fig. 5a) is listed in Table 1. The C-terminal his_6_-tagged ERM peptide produced in *E. coli* NovaBlue (DE3) was purified by Chelating Sepharose Fast Flow (GE Healthcare), and then used to generate the polyclonal rabbit anti-ERM antiserum (AngeneBiotech, Taipei).

### Plasmid DNA transfection

The plasmid RCAS-myrAkt (Addgene) was introduced into RAW 264.7 cells by transfection using X-tremeGENE HP DNA Transfection Reagent (Roche). The cells were subcultured for further analysis after incubation for 48 h to 72 h.

### Immunoblotting

The proteins in total cell lysate or immunocomplexes were fractionated by 6% SDS-polyacrylamide gel electrophoresis and then transferred to a PVDF membrane. The membrane was hybridized with primary antibodies (Abs) followed by horse radish peroxidase-conjugated secondary Abs, and the hybridized bands were visualized by enhanced chemiluminescence (PerkinElmer). Densitometric analysis of Akt Pi-S473 was performed with ImageJ software and the results were normalized to total protein level of Akt. The Abs against Pyk2, Pyk2 Pi-Y402, SFKs, PI3K p85 Pi-Y458/p55 Pi-Y199, Akt, Akt Pi-S473, p38 and p38 Pi-T180/Y182 were purchased from Cell Signaling Technology. Abs against FAK Pi-Y861 and PI3K p85 were purchased from GenScript. Abs against SFKs Pi-Y418, actin and FAK were purchased from Invitrogen, Millipore and BD Biosciences, respectively. Abs against Lyn, Fgr and Hck were purchased from Santa Cruz.

### Immunoprecipitation

One ml of whole cell lysate (containing 2–5 mg proteins) was pre-cleared by protein G agarose beads (Millipore), and then mixed with 1.5 μg of relevant Abs. After incubation at 4 °C overnight, the immunocomplexes were captured by protein G agarose beads, washed and denatured by boiling. The proteins in the immunocomplexes were then detected by immunoblotting.

### Statistical analyses

Paired Student’s *t*-tests (two-tailed) and one-way analysis of variance (ANOVA) followed by Tukey’s test were performed with Prism 5.01 (GraphPad Software).

## Results

### Effect of MARTX_Vv1_ on morphological change of and actin polymerization in infected macrophages

**Fig. 1:**
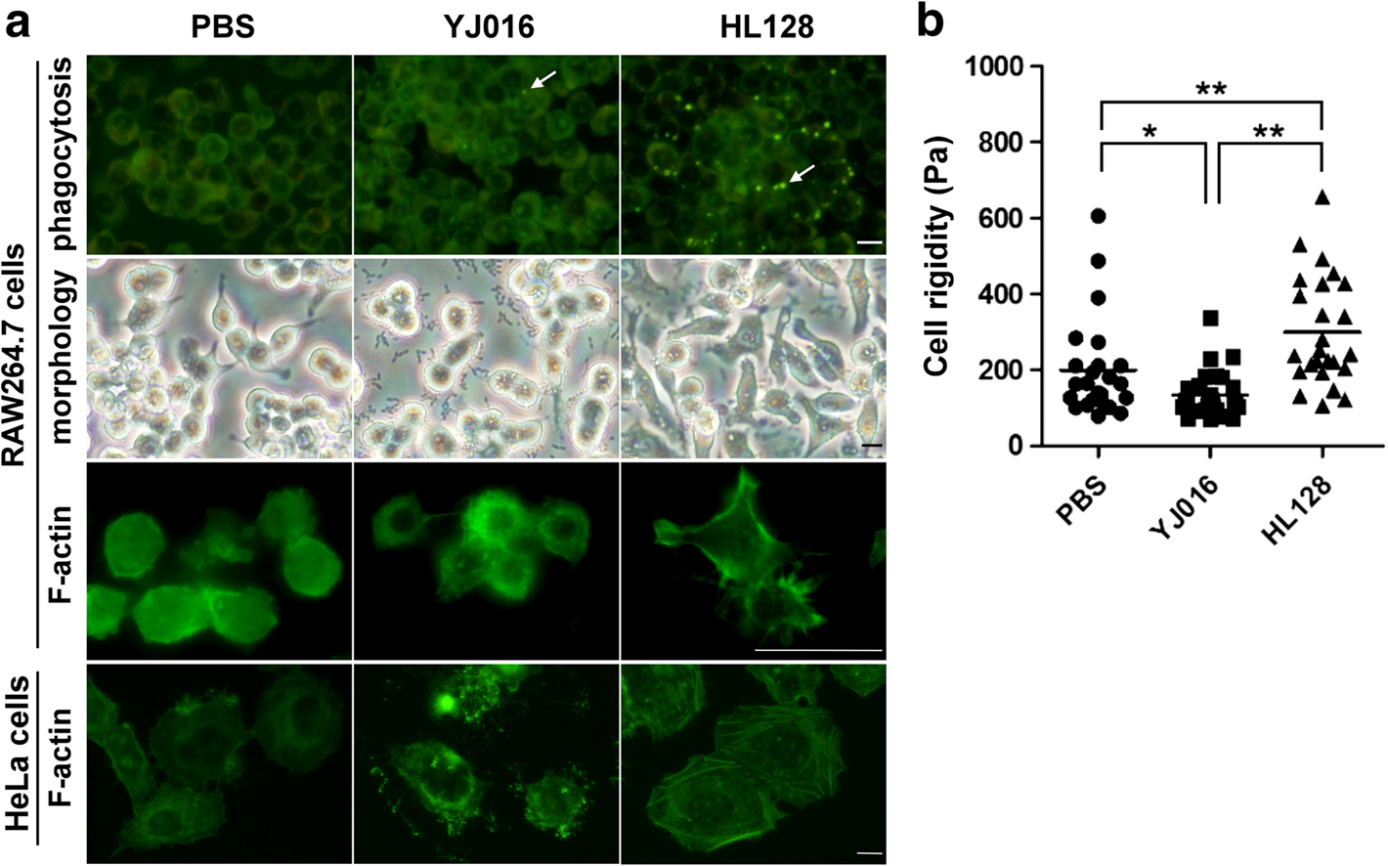
Morphology, rigidity, F-actin organization and bacteria engulfment of the infected cells. **a** RAW 264.7 or HeLa cells were coincubated with the WT strain (YJ016) and the MD mutant (HL128) at MOI 10 for 90 min. The morphology of the cells was then examined under a light microscope. The engulfed bacteria (indicated by arrows) were detected under a fluorescence microscope after acridine orange-crystal violet stain. F-actin was stained by Alexa Fluor® 488 conjugated phalloidin and observed by fluorescence microscopy. Bar = 20 μm. **b** Rigidity of RAW 264.7 cells examined by AFM is expressed as Young’s Modulus (Pa). *: *p* < 0.05 and **: *p* < 0.01 by paired Student’s *t*-tests (two-tailed). *n* = 3

**Fig. 2:**
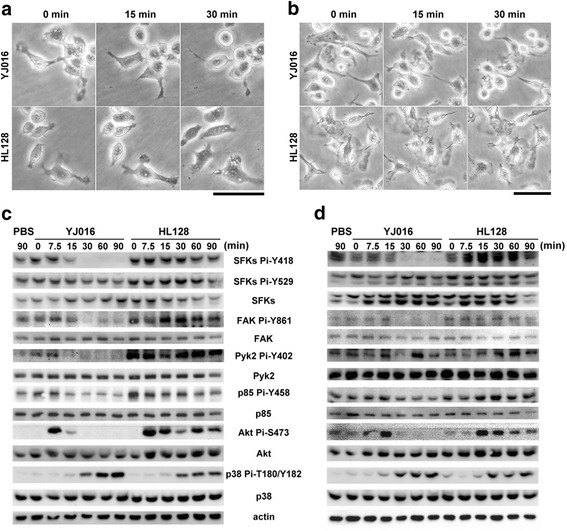
Morphological change and phosphorylation levels of phagocytosis-related kinases of macrophages after infection by WT strain or MD mutant. RAW 264.7 cells (**a**, **b**) and mPEM (**b**, **d**) were coincubated with the WT strain (YJ016) or MD mutant (HL128) at MOI 10 for the indicated periods. **a**, **b** The morphological change of infected cells was examined by time-lapse microscopy. Bar = 100 μm. **c**, **d** The infected cells were lysed, and the total amount and phosphorylation level at the indicated amino acid residue(s) of each kinase were estimated by immunoblotting with relevant antibodies. Data are representative of three independent experiments

Infection of the MARTX _Vv1_-deficient (MD) mutant, HL128, caused formation of stress fibers in the HeLa cells and pseudopodia in the RAW 264.7 cells (Fig. 1a). However, infection of the MARTX_Vv1_-producing WT strain resulted in cell rounding of both cell lines and apparent F-actin disorganization in the HeLa cells (Fig. 1a) 90 min after infection. In addition, by AFM we detected reduced rigidity of RAW 264.7 cells 90 min after infection by the WT strain but increased rigidity of those infected by the MD mutant (Fig. 1b). By time-lapse microscopy, we found that RAW 264.7 cells infected by the WT strain, but not MD mutant, lost pseudopodia and rounded up starting from 15 min after infection (Fig. 2a).

It has been demonstrated that MARTX_Vv1_ exerts comparable cytotoxicity and antiphagocytosis effect in the RAW 264.7 cells and mouse peritoneal exudate macrophages (mPEM) [10]. We further tested whether mPEM may undergo cell rounding in the presence of MARTX_Vv1_. As shown in Fig. 2b, like RAW 264.7 cells, mPEM infected by the WT strain, but not the MD mutant, rounded up starting from 15 min after infection.

### Effect of MARTX_Vv1_ on phosphorylation levels of phagocytosis-related kinases

To determine the effect of MARTX_Vv1_ on the signaling molecules that regulate actin polymerization, we checked the phosphorylation levels of SFKs, FAK, Pyk2, PI3K, Akt and p38 MAP kinase in RAW 264.7 cells after infection by the WT strain and MD mutant. We found that the WT strain caused dephosphorylation at SFKs Y418, FAK Y861, Pyk2 Y402 and PI3K p85 Y458, but not SFKs Y529 or p38 T180/Y182, 7.5 min or 15 min after infection at MOI 10 (Fig. 2c).

Phosphorylation of Akt at Ser473 was detected at 7.5 min after infection by either strain, but it was then dramatically reduced to an undetectable level from 15 min after infection by the WT strain, but not the MD mutant (Fig. 2c). The reduced phosphorylation levels of these proteins in the presence of MARTX_Vv1_ were not due to decreased protein expression, because the total amount of each protein was not affected (Fig. 2c). The WT strain also caused dephosphorylation at SFKs Y418, FAK Y861, Pyk2 Y402, PI3K p85 Y458 and Akt S473, but not SFKs Y529 and p38 T180/Y182, in mPEM (Fig. 2d).

**Fig. 3:**
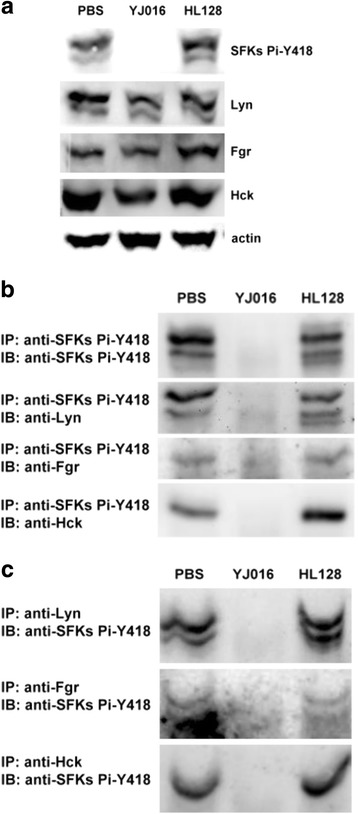
Phosphorylation levels of Src family members, Lyn, Fgr and Hck, in infected macrophages. The total cell lysate of RAW 264.7 cells coincubated with the WT strain (YJ016) or MD mutant (HL128) at MOI 10 for 90 min was collected and subjected to immunoblotting (**a**) and immunoprecipitation (**b**, **c**). In **b** and **c**, the phosphorylated proteins were immunoprecipitated (IP) or detected by immunoblotting (IB) with the indicated antibodies. Data are representative of three independent experiments

As Lyn, Fgr and Hck are known to be the predominant SFKs in macrophages [40, 41], we further determined which of them was dephosphorylated after infection by the WT strain. The protein level of each of them was not significantly affected in the presence of MARTX_Vv1_ (Fig. 3a). We then performed immunoprecipitation to determine the phosphorylation level of each protein. In one experiment, precipitation of all SFKs that were phosphorylated at Tyr418 with anti-SFKs Pi-Y418 Ab was followed by immunoblotting with Ab against Lyn, Fgr or Hck to detect each protein (Fig. 3b). In another experiment, precipitation of both the phosphorylated and unphosphorylated proteins with anti-Lyn, -Fgr or -Hck Ab was followed by immunoblotting with anti-SFKs Pi-Y418 Ab to detect the phosphorylated molecules (Fig. 3c). In either experiment we found that Tyr418 of Lyn, Fgr and Hck were all phosphorylated in the absence of MARTX_Vv1_, but were all dephosphorylated in the presence of MARTX_Vv1_, in RAW 264.7 cells 90 min after infection.

### Effects of SFKs, FAK/Pyk2, PI3K and Akt inhibitors on engulfment of MD mutant and phosphorylation of signaling molecules in infected macrophage

**Fig. 4:**
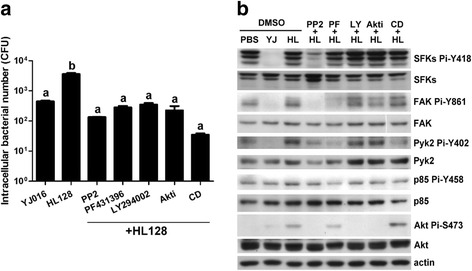
Effects of specific inhibitors of phagocytosis-related kinases on phagocytosis and phosphorylation of these kinases in infected macrophages. RAW 264.7 cells were pretreated with PP2 (SFKs inhibitor), PF-431396 (Pyk2/FAK inhibitor, PF), LY294002 (PI3K inhibitor, LY), Akt1/2 kinase inhibitor (Akti) and cytochalasin D (F-actin inhibitor, CD) for 4 h, 1 h, 1 h, 1 h and 1 h, respectively. Phagocytosis (**a**) and the total amount as well as phosphorylation level of each signaling molecule (**b**) were then estimated by gentamycin protection assay and immunoblotting, respectively, 90 min after coincubation of the pretreated cells with the bacteria at MOI 10. YJ: YJ016, WT strain; HL: HL128, MD mutant. DMSO: 5% dimethyl sulfoxide, the solvent used as negative control. Data in **a** were analyzed by one-way ANOVA followed by Tukey’s test. *n* = 3. Bars labeled with ‘a’ are not significantly different from each other, but are significantly different from that labeled with ‘b’ (*P* < 0.05). Data in **b** are representative of three independent experiments

We further used the inhibitors of SFKs, FAK/Pyk2, PI3K and Akt to determine the involvement of these molecules and the relations among them in engulfment of the MD mutant by the macrophage. As shown in Fig. 4a, all of these inhibitors significantly impaired the phagocytosis of MD mutant by RAW 264.7 cells. In addition, the inhibitor of SFKs (PP2) reduced the phosphorylation levels at not only SFKs Y418 but also FAK Y861, Pyk2 Y402 and Akt S473 (Fig. 4b), namely, inactivation of SFKs caused dephosphorylation of FAK, Pyk2 and Akt. The inhibitor of FAK and Pyk2 (PF431396) reduced the phosphorylation levels at FAK Y861, Pyk2 Y402, PI3K p85 Y458 and Akt S473 (the band intensity of Akt Pi-S473 of the treated cells was 68% of that of the untreated cells), but not SFKs Y418. This shows that inactivation of FAK and Pyk2 can cause dephosphorylation of PI3K and Akt, but not SFKs. The inhibitors of PI3K (LY294002) and Akt (Akt1/2 inhibitor) reduced the phosphorylation level of only Akt S473 (Fig. 4b), indicating that inactivation of PI3K can cause dephosphorylation of Akt, but not SFKs, FAK or Pyk2. More, inactivation of Akt could not cause dephosphorylation of all the other tested kinases. The F-actin inhibitor, cytochalasin D, although significantly inhibited the internalization of MD mutant (Fig. 4a), did not affect the phosphorylation levels of these kinases except for Pyk2 Y402 (Fig. 4b).

### Involvement of MARTX_Vv1_ effector domains in cytotoxicity and antiphagocytosis

**Fig. 5:**
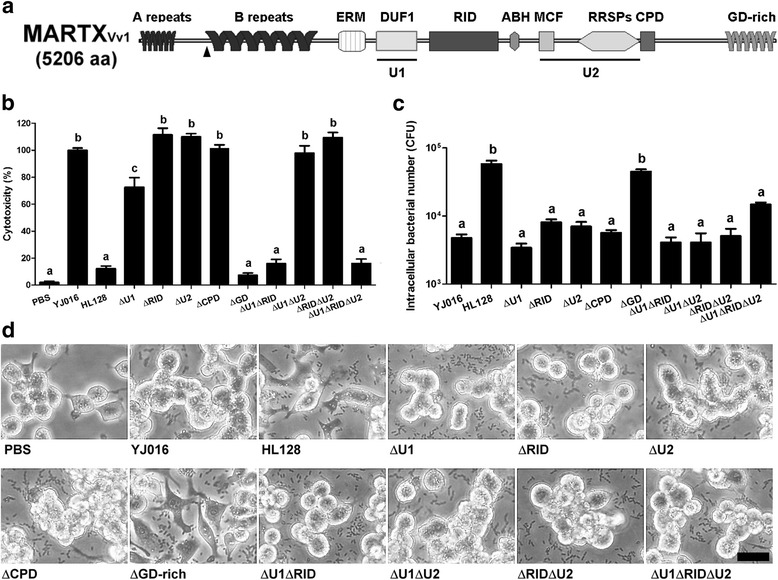
Cytotoxicity, antiphagocytosis activity and ability to cause cell rounding of the MARTX_Vv1_ domain-deletion mutants. **a** The effector domains in MARTX_Vv1_. The triangle indicates the 469-bp deletion in the MD mutant, HL128, that results in a frameshift leading to a stop codon 26 bp downstream of the deletion. **b**, **c**, and **d** The bacteria were cultured in LB for 4 h, washed, and coincubated with RAW 264.7 cells at MOI 10 for 3 h (**b**) or 90 min (**c**, **d**). The cytotoxicity (**b**) and internalized bacterial number (**c**) were then estimated by LDH assay and gentamycin protection assay, respectively. The cell morphology (**d**) was examined under a light microscope. Bar = 50 μm. YJ016: WT strain; HL128: MD mutant. Data in **b**, and **c** were analyzed by one-way ANOVA followed by Tukey’s test. *n* = 3. Bars that show no significant difference from each other are labeled with the same letter, and those showing significant difference (*P* < 0.05) are labeled with different letters. Data in **d** are representative of three independent experiments

To determine the roles of various domains of MARTX_Vv1_ (Fig. 5a) in antiphagocytosis and lysis of macrophages, we isolated mutants with single, double or triple in-frame deletions of unique sequence 1 (U1, containing DUF1), RID, unique sequence 2 (U2, containing MCF and RRSPs), CPD and GD-rich domain. These mutants all expressed the MARTX_Vv1_ mutant proteins (Additional file 1: Figure S1).

**Fig. 6:**
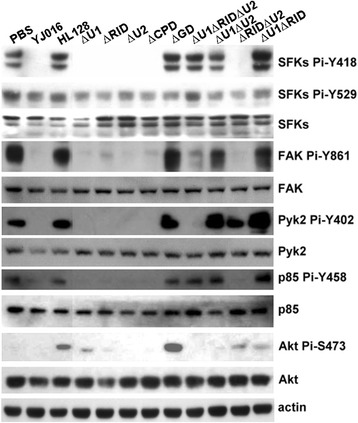
Phosphorylation levels of phagocytosis-related kinases in macrophages infected by MARTX_Vv1_ domain-deletion mutants. The bacteria were cultured in LB for 4 h, washed, and coincubated with RAW 264.7 cells at MOI 10 for 90 min. Total cell lysate of the infected RAW 264.7 cells was collected, and the phosphorylation level of each kinase was examined by immunoblotting. YJ016: WT strain; HL128: MD mutant. Data are representative of three independent experiments

Deletion of RID, U2 or CPD domain alone and deletion of both RID and U2 showed wild-type level of cytotoxicity (Fig. 5b) and antiphagocytosis activity (Fig. 5c). Deletion of U1 although resulted in a slight decrease of cytotoxicity to 72.5% of that of the WT strain, it did not affect the antiphagocytosis activity. In addition, the ability of all of these mutants to cause cell rounding (Fig. 5d) and dephosphorylation of phagocytosis-related kinases in RAW 264.7 cells were not significantly different from that of the WT strain (Fig. 6). On the contrary, deletion of the GD-rich repeats domain eliminated all of these MARTX_Vv1_-mediated activities (Fig. 5) and the ability to cause dephosphorylation of the phagocytosis-related kinases (Fig. 6).

The Δ*u1*Δ*rid* double and Δ*u1*Δ*rid* Δ*u2* triple mutants although exhibited reduced cytotoxicity (Fig. 5b), they caused cell rounding (Fig. 5d) and their antiphagocytosis activity was normal (Fig. 5c). More, the MARTX_Vv1_-mediated dephosphorylation of phagocytosis-related kinases was abolished in cells infected by these two mutants, except for Akt S473 in the Δ*u1*Δ*rid* mutant-infected and Akt S473 as well as FAK Y861/Pyk Y402 in the Δ*u1*Δ*rid* Δ*u2* mutant-infected cells (Fig. 6). The Δ*u1*Δ*u2* mutant although resembled the WT strain in cytotoxicity, antiphagocytosis activity and ability to cause cell rounding (Fig. 5b-d), the phosphorylation levels of the phagocytosis-related kinases, except for Akt S473, was not decreased in cells infected by this mutant (Fig. 6).

### Effect of myrAkt on phagocytosis of macrophages

**Fig. 7:**
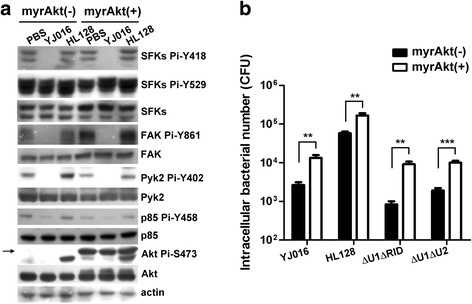
Effects of myrAkt on phagocytosis and phosphorylation levels of phagocytosis-related kinases in macrophages. The normal 〔myr-Akt(−)〕 and myrAkt-expressing 〔myr-Akt(+)〕 RAW 264.7 cells were coincubated with the washed bacteria at MOI 10 for 90 min. The total amount and phosphorylation level at the indicated amino acid residue(s) of each kinase (**a**) as well as the internalized bacteria number (**b**) were then estimated by immunoblotting and gentamycin protection assay, respectively. The band of myrAkt is indicated by an arrow. YJ016: WT strain; HL128: MD mutant. **: *P* < 0.01; ***: *P* < 0.001 by paired Student’s *t*-tests (two-tailed). *n* = 3. Data in **a** are representative of three independent experiments

We showed that MARTX_Vv1_ could cause dephosphorylation of SFKs Y418, FAK Y861, Pyk2 Y402, PI3K p85 Y458 and Akt S473, and inactivation of Akt alone resulted in reduced phagocytosis in RAW 264.7 cells (Fig. 4). In addition, decreased phosphorylation level at Akt S473 correlated well with impaired phagocytosis of RAW 264.7 cells (Fig. 5c and 6). To confirm the role of Akt in phagocytosis of *V. vulnificus*, we tested whether expression of the constitutively active myrAkt [42, 43] in RAW 264.7 cells could result in increased phagocytosis of this organism. As shown in Fig. 7a, phosphorylated myrAkt was expressed in cells with or without infection, while the phosphorylation levels of SFKs, FAK, p85 of PI3K and Akt were not significantly affected. Compared to the normal cells, those expressing myrAkt exhibited significantly increased phagocytosis of the WT strain or MARTX_Vv1_ domain-deletion mutants, Δ*u1*Δ*rid* and Δ*u1*Δ*u2*, which showed wild-type antiphagocytosis ability (Fig. 7b).

### Involvement of effector domains in MARTX_Vv1_-mediated cytotoxicity and cell rounding in macrophages and epithelial cells

*V*. *vulnificus* may encounter different types of cells, including the epithelial cells and phagocytes, during infection. To determine whether the central region of MARTX_Vv1_ is similarly involved in the MARTX_Vv1_-mediated cytotoxicity and cell rounding in different cell types, we examined the HeLa and RAW 264.7 cells infected by the Δ*u1*Δ*rid* and Δ*u1*Δ*rid* Δ*u2* mutants for these properties. To avoid the effect of cytolysin, which can cause cell lysis in the bacteria-cell coincubation system at high MOI or after prolonged incubation [17], we deleted its gene, *vvhA*, from all the tested strains.

**Fig. 8:**
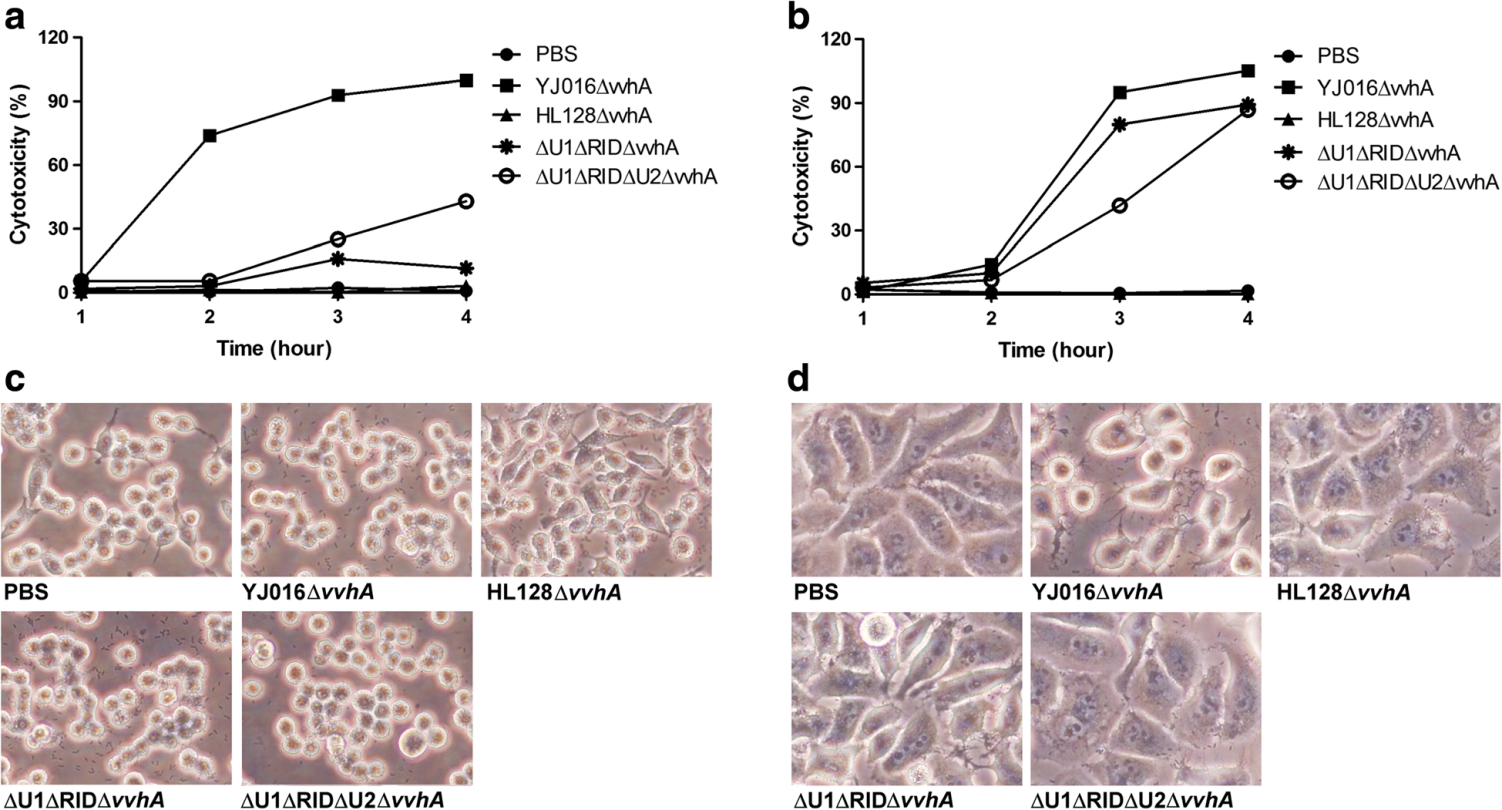
MARTXVv1-mediated cytotoxicity and morphological change in macrophage and epithelial cell lines**.** The bacteria were cultured in LB for 4 h, washed, and coincubated with RAW 264.7 cells (**a**, **c**) or HeLa cells (**b**, **d**) at MOI 10 for the indicated periods (**a**, **b**) or 1 h (**c**, **d**). The cytotoxicity (**a**, **b**) was estimated by LDH assay (*n* = 3), and the cell morphology (**c**, **d**) was examined under a light microscope. YJ016: WT strain; HL128: MD mutant. Data in **c** and **d** are representative of three independent experiments

The WT strain, but not the MD mutant, caused cytotoxicity of RAW 264.7 cells and HeLa cells 2 h (Fig. 8a) and 3 h (Fig. 8b), respectively, after infection. The cytotoxicities of Δ*u1*Δ*rid* and Δ*u1*Δ*rid* Δ*u2* mutants to RAW 264.7 cells were much lower than that of the WT strain up to 4 h post infection (Fig. 8a), while their cytotoxicity to HeLa cells were comparable to the WT strain (Fig. 8b). In addition, the WT strain, but not the MD mutant, caused rounding of both RAW 264.7 and HeLa cells 1 h after infection (Fig. 8c and d). However, the Δ*u1*Δ*rid* and Δ*u1*Δ*rid* Δ*u2* mutants although caused rounding of RAW 264.7 cells (Fig. 8c), they did not cause rounding of HeLa cells (Fig. 8d), even up to 3.5 h post infection (data not shown).

We also found that the Δ*rid*, Δ*gd*, Δ*u1*Δ*rid* , Δ*rid* Δ*u2* and Δ*u1*Δ*rid* Δ*u2* mutants, like the MD mutant, did not cause rounding of the HeLa cells 90 min after infection, while the Δ*u1*, Δ*u2*, Δ*cpd* and Δ*u1*Δ*u2* mutants, like the WT strain, caused rounding of these cells (Additional file 2: Figure S2).

## Discussion

MARTX is the major cytotoxin of *V. vulnificus*, and it causes lysis of various mammalian cells, including macrophages and epithelial cells. We have previously demonstrated that the biotype 1 *V. vulnificus* strain, YJ016, produces MARTX_Vv1_ to protect itself from engulfment by the macrophage before the phagocyte is lysed by this cytotoxin [10]. In this study, we explored how MARTX_Vv1_ exerts the antiphagocytosis effect on macrophages by comparing between the macrophages infected by the WT strain and those infected by the MD mutant for a variety of phagocytosis-related properties. We found that the mouse macrophage cell line, RAW 264.7, and mPEM incubated with either the WT strain or MD mutant formed pseudopodia shortly after infection (Fig. 2a and b). But, the WT strain-infected macrophages, which internalized much fewer bacteria (Fig. 1a), began to round up around 15 min after incubation, while those infected by the MD mutant remained adherent and active (Fig. 2a and b). In addition, the RAW 264.7 cells became more rigid after infection by the MD mutant, but they became less rigid, suggesting F-actin depolymerization as proposed previously [44], after infection by the WT strain (Fig. 1b). HeLa cells exhibited morphological change similar to that of RAW 264.7 cells after infection by either strain. Nevertheless, the stress fibers, which were disintegrated and became aggregates in the rounded cells 90 min after infection by the WT strain, were clearly observed in HeLa cells, but not RAW 264.7 cells (Fig. 1a). These suggest that MARTX_Vv1_ might inhibit cytoskeleton rearrangement in the macrophage to result in cell rounding and inability to internalize the infecting bacteria.

Actin rearrangement in macrophage during phagocytosis is regulated by a number of signaling molecules, which are activated in a cascade by phosphorylation at specific amino acid residues [32, 34, 35, 45–47] when the signal is triggered upon bacterium-macrophage interaction. In both the RAW 264.7 cells and mPEM, infection by the WT strain, but not MD mutant, resulted in dephosphorylation at SFKs Y418, FAK Y861, Pyk2 Y402, PI3K p85 Y458 and Akt S473 (Fig. 2c and d). This suggests that MARTX_Vv1_ might interfere with phagocytosis by inactivating these kinases. The p38 MAP kinase has been shown to be involved in the TLRs-MyD88 pathway-mediated phagocytosis of interacting bacteria [35]. Nevertheless, antiphagocytosis mediated by MARTX_Vv1_ may not be via inactivating this TLR-dependent signaling pathway, because T180/Y182 of p38 was phosphorylated upon infection despite the presence of MARTX_Vv1_ (Fig. 2c and d).

MARTX_Vv1_ caused dephosphorylation of all the predominant SFKs, i.e. Lyn, Fgr and Hck, in RAW 264.7 cells (Fig. 3). These SFK members have been shown to be associated with different receptors residing in the lipid raft [48], a specific plasma membrane platform for organizing the receptors and their downstream molecules to initiate signaling pathways [49]. The possibility that MARTX_Vv1_ may cause dephosphorylation of SFKs by disrupting the lipid rafts was excluded as this structure remained intact up to 90 min after infection by the WT strain (our unpublished data). Dephosphorylation at Y418 of SFKs might not due to a negative feedback on the autokinase activity resulting from increased phosphorylation at Tyr529 of SFKs [45], as phosphorylation of Tyr529 was in fact slightly reduced in cells infected by the WT strain (Fig. 2c and d). Whether phosphatases may be involved in dephosphorylation of SFKs awaits further investigation.

By using the kinase inhibitors of SFKs, FAK/Pyk2, PI3K and Akt, we further demonstrated that these kinases were all involved in phagocytosis of the MD mutant (Fig. 4a). In addition, from the effect of each inhibitor on the phosphorylation levels of these molecules (Fig. 4b), a signaling pathway, SFKs-FAK/Pyk2-PI3K-Akt, can be depicted. The order of these molecules in this signaling pathway is supported by other studies, which showed that FAK and Pyk2 were phosphorylated by the activated SFKs [50–52]; PI3K was downstream of FAK [53] and Pyk2 [51]; and Akt was activated by the phosphorylated PI3K [54]. Like treatment with the SFKs inhibitor, PP2, infection by the WT strain resulted in dephosphorylation of these molecules, suggesting that MARTX_Vv1_ might cause inactivation of this signaling pathway to impair bacteria engulfment.

There are several putative effector domains in the central region of MARTX_Vv1_, and some of them share high similarity to their equivalents in the MARTX of *V. cholerae*. By characterizing a set of MARTX_Vv1_ effector domain-deletion mutants, we showed that domains DUF1, RID and CPD as well as the U2 region that contains MCF and RRSPs were individually dispensable for the MARTX_Vv1_-mediated cytotoxicity, cell rounding, antiphagocytosis and dephosphorylation of the phagocytosis-related kinases in the macrophage (Fig. 5b-5d, Fig. 6). On the contrary, deletion of the GD-rich repeats domain abolished these effects of MARTX_Vv1_ (Fig. 5b-5d, Fig. 6) as was expected based on the crucial role of this domain in secretion of this toxin [13].

Interestingly, Δ*u1*Δ*rid* and Δ*u1*Δ*rid*Δ*u2*, showed markedly reduced cytotoxicity but exhibited wild-type antiphagocytosis activity and caused cell rounding (Fig. 5b-5d). This is different from the results of a study conducted with the epithelial cell line, HeLa cells. In that study, Kim et al. found that the effector domains-containing central region of MARTX_Vv1_ was associated with the ability to cause cell rounding, but not cell lysis [16]. Here we show that the Δ*u1*Δ*rid* and Δ*u1*Δ*rid*Δ*u2* mutants displayed wild-type cytotoxicity to HeLa cells but, unlike the WT strain, they did not cause rounding of these cells (Fig. 8b and d). However, in RAW 264.7 cells these two mutants although exhibited low cytotoxicity, they caused cell rounding (Fig. 8a and c). These indicate that the epithelial cell and macrophage responded differently to these two MARTX_Vv1_ mutant proteins, with the former being more readily lysed and the later more easily rounding up. We confirmed that RID, which inactivates Rho, Rac and Cdc42 to result in actin depolymerization [19], is required for rounding of the epithelial cells mediated by MARTX_Vv1_, since all and only the mutants with deletion of RID lost the ability to cause rounding of HeLa cells (Additional file 2: Figure S2). However, deletion of the majority part of central region in the Δ*u1*Δ*rid*Δ*u2* mutant did not result in loss of the ability to cause cell rounding and inhibit phagocytosis of RAW 264.7 cells (Fig. 5c and d). The N-and C-termini of this toxin, instead, might be responsible for causing cell rounding and inhibition of phagocytosis in the macrophage by a mechanism that awaits further exploration. It is not clear at the moment why these two types of cell responded differently to the MARTX_Vv1_ domain-deletion mutants.

The defect of the Δ*u1*Δ*rid* and Δ*u1*Δ*rid*Δ*u2* mutants in cytotoxicity to RAW 264.7 cells may result from reduced amount and/or activity of these MARTX_Vv1_ mutant proteins interacting with the cells, which remains to be determined. In addition, as these two mutants were defective in cytotoxicity, but not antiphagocytosis, it suggests that MARTX_Vv1_ may execute cytotoxicity and antiphagocytosis by different mechanisms.

Our data clearly indicate that the ability of the WT strain and MARTX_Vv1_ domain-deletion mutants to inhibit phagocytosis correlated with their ability to cause rounding of the macrophage. Intriguingly, the Δ*u1*Δ*rid*, Δ*u1*Δ*u2* and Δ*u1*Δ*rid*Δ*u2* mutants although retained the antiphgocytosis activity and ability to cause cell rounding (Fig. 5c and d), they did not cause dephosphorylation of the tested kinases, except Akt for the Δ*u1*Δ*rid* and Δ*u1*Δ*u2* mutants and FAK/Pyk2, in addition, for the Δ*u1*Δ*rid*Δ*u2* mutant (Fig. 6). The Δ*u1*Δ*rid*Δ*u2* mutant, which resulted in complete dephosphorylation of Akt, showed slightly lower antiphagocytosis activity (without statistically significant difference) compared to the Δ*u1* mutant, which resulted in partial dephosphorylation of Akt (Fig. 5c and 6). This could be because the Δ*u1*Δ*rid*Δ*u2* mutant was less functional than the Δ*u1* mutant due to a larger deletion region in MARTX_Vv1_. We further found that engulfment of the WT strain as well as the Δ*u1*Δ*rid* and Δ*u1*Δ*u2* mutants was increased significantly in RAW 264.7 cells that expressed the constitutively active Akt, myr-Akt (Fig. 7). Collectively, our data suggest that a reduced phosphorylation level at Akt S473 might be responsible for inhibition of phagocytosis by MARTX_Vv1_, which is plausible as Akt has been shown to physically interact with the actin filament [55]. Nevertheless, engulfment of the WT strain or the Δ*u1*Δ*rid* and Δ*u1*Δ*u2* mutants was not restored to the level of MD mutant-infected cells in the presence of myr-Akt, implying that there may be other MARTX_Vv1_-mediated antiphagocytosis mechanisms. Alternatively, myr-Akt, although is phosphorylated, may not be as active as the phosphorylated endogenous Akt. Akt may not inhibit phagocytosis by directly inactivating actin polymerization, because inhibition of actin polymerization by cytochalasin D did not affect the phosphorylation levels of these signaling molecules (Fig. 4b).

## Conclusions

Our data show that MARTX_Vv1_ of *V. vulnificus* causes inactivation of the phagocytosis-related SFKs-FAK/Pyk2-PI3K-Akt signaling pathway, which could lead to cell rounding and impaired phagocytosis of the infected macrophage. In addition, the majority of the central region, which consists of the effector domains, of MARTX_Vv1_ is not associated with the antiphagocytosis activity.

## Additional files

Additional file 1: Figure S1. Expression of MARTX_Vv1_ mutant proteins in the domain-deletion mutants. Total cell lysate collected from the bacteria cultured in LB for 4 h was fractionated by electrophoresis on an 8% SDS-polyacrylamide gel and then subjected to immunoblotting with anti-ERM antibody. YJ016: WT strain; HL128: MD mutant. Data are representative of three independent experiments. (TIFF 989 kb)

Additional file 2: Figure S2. Morphological change of HeLa cells infected by various MARTX_Vv1_ domain-deletion mutants. Morphology of the HeLa cells coincubated with bacteria at MOI 10 for 90 min was examined under a light microscope. YJ016: WT strain; HL128: MD mutant. Bar = 50 μm. Data are representative of three independent experiments. (TIFF 3870 kb)

## Abbreviations

Ab: Antibody
LB medium: Luria-Bertani medium
MOI: Multiplicity of infection
PCR: Polymerase chain reaction
