# Supplementary figures and images for: Vibrio vulnificus MARTX cytotoxin causes inactivation of phagocytosis-related signaling molecules in macrophages
Source: J Biomed Sci. 2017 Aug 19;24:58. doi: 10.1186/s12929-017-0368-2 (PMC5563386; doi:10.1186/s12929-017-0368-2)

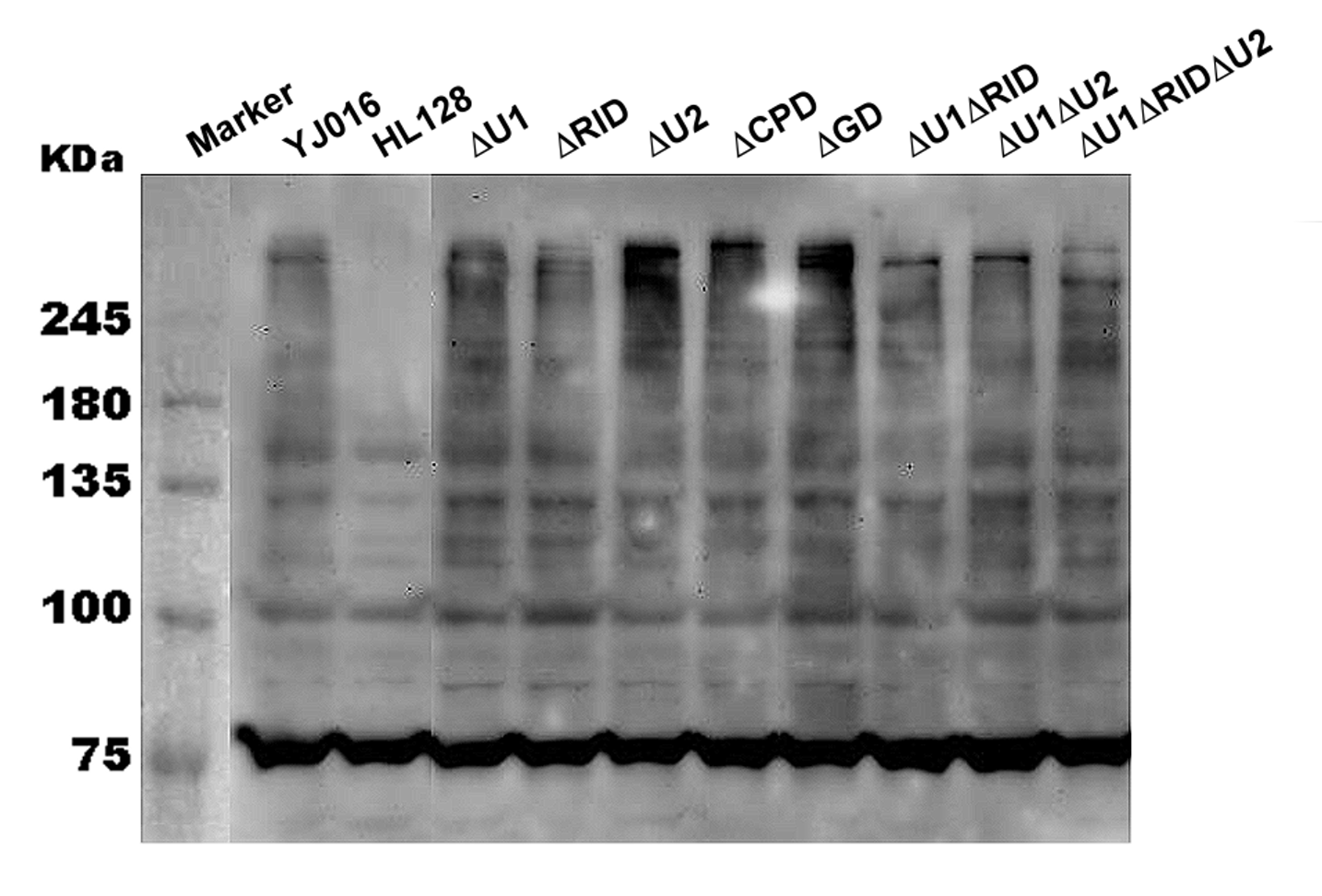

Supplement: Supplementary file 1 — Expression of MARTXVv1 mutant proteins in the domain-deletion mutants. Total cell lysate collected from the bacteria cultured in LB for 4 h was fractionated by electrophoresis on an 8% SDS-polyacrylamide gel and then subjected to immunoblotting with anti-ERM antibody. YJ016: WT strain; HL128: MD mutant. Data are representative of three independent experiments. (TIFF 989 kb) [file 12929_2017_368_MOESM1_ESM.tif]

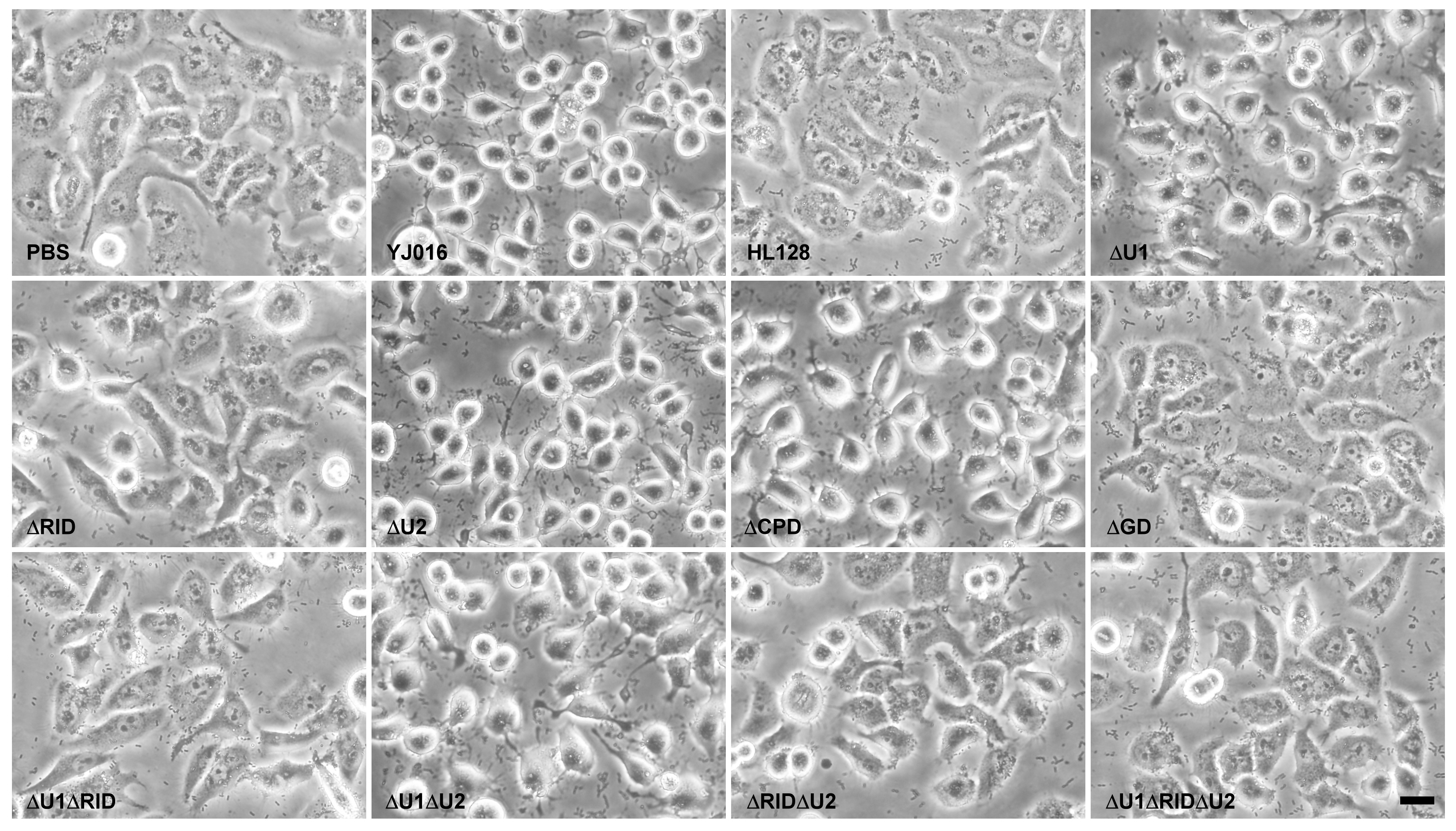

Supplement: Supplementary file 2 — Morphological change of HeLa cells infected by various MARTXVv1 domain-deletion mutants. Morphology of the HeLa cells coincubated with bacteria at MOI 10 for 90 min was examined under a light microscope. YJ016: WT strain; HL128: MD mutant. Bar = 50 μm. Data are representative of three independent experiments. (TIFF 3870 kb) [file 12929_2017_368_MOESM2_ESM.tif]
